# A Novel Blend of *Momordica charantia* and *Stevia rebaudiana* Extracts Ameliorates Metabolic Dysfunction and Muscle Atrophy in Type 2 Diabetic Mice

**DOI:** 10.3390/foods15132364

**Published:** 2026-07-03

**Authors:** Ji-Hwan Yoon, Varun Jaiswal, Miey Park, Hae-Jeung Lee

**Affiliations:** 1 Department of Food and Nutrition, College of BioNano Technology, Gachon University, Sujeong-gu, Seongnam-si 13120, Gyeonggi-do, Republic of Korea; yoonjw1026@gachon.ac.kr (J.-H.Y.); computationalvarun@gmail.com (V.J.); 2 Institute for Aging and Clinical Nutrition Research, Gachon University, Sujeong-gu, Seongnam-si 13120, Gyeonggi-do, Republic of Korea; 3 Department of Health Sciences and Technology, Gachon Advanced Institute for Health Science and Technology (GAIHST), Gachon University, 155 Gaetbeol-ro, Yeonsu-gu, Incheon 21999, Republic of Korea

**Keywords:** T2DM, *M. charantia*, *Stevia rebaudiana*, *Barnesiella intestinihominis*

## Abstract

Type 2 diabetes mellitus (T2DM) involves progressive muscle wasting, metabolic dysregulation in peripheral tissues, chronic hyperglycemia, and insulin resistance. *Momordica charantia* is an antidiabetic agent often limited by bitterness. To improve palatability and efficacy, we developed EMS by combining *M. charantia* and *Stevia rebaudiana* (9:1). EMS’s antidiabetic effects were tested in streptozotocin (STZ)-induced and genetic *db*/*db* mouse models of diabetes. Mice received oral EMS at doses (40, 80, and 120 mg/kg) for six weeks, assessing glucose tolerance, insulin sensitivity, lipid profile, and hepatic markers. Additionally, muscle protein synthesis and degradation mechanisms were analyzed in gastrocnemius tissues. EMS significantly reduced fasting blood glucose and improved insulin sensitivity in both models. EMS decreased liver lipid accumulation and serum ALT and AST levels, indicating hepatic protection. EMS alleviated muscle atrophy by increasing muscle fiber area and was associated with increased expression or activity of AMPK/Sirt1/PGC-1α and IRS-1/PI3K/AKT insulin pathways. It also suppressed FOXO3a-mediated expression of Atrogin-1 and MuRF1, suggesting reduced activation of protein-degradation pathways. Moreover, EMS modulated the gut microbiota, increasing the abundance of beneficial species such as *Barnesiella intestinihominis*. These findings suggest EMS is a promising multitarget functional ingredient for metabolic complications and musculoskeletal decline in T2DM.

## 1. Introduction

The global burden of type 2 diabetes mellitus (T2DM) has increased sharply over the past few decades and emerged as a major public health concern [1]. T2DM is characterized by impaired insulin secretion and a progressive decline in insulin sensitivity [2]. Genetic predisposition, along with lifestyle-related factors such as excessive caloric intake, obesity, and physical inactivity, is a major contributor to disease development [3]. These metabolic abnormalities result in chronic hyperglycemia, often accompanied by complications, including dyslipidemia, inflammation, and oxidative stress [4].

As the disease progresses, insulin resistance intensifies, leading to reduced cellular glucose uptake and persistent hyperglycemia despite normal or elevated insulin levels [5]. Unlike type 1 diabetes, which is caused by the destruction of pancreatic β-cells due to autoimmunity, T2DM is characterized by chronic insulin resistance and a gradual decline in β-cell function [6,7]. In addition, the decline in β-cell function is accompanied by morphological changes in the pancreas, especially a decrease in islet size [8]. This deterioration in β-cell function, together with peripheral insulin resistance, leads to impaired insulin secretion and widespread metabolic dysregulation in peripheral tissues [9]. Impaired insulin signaling reduces glucose utilization, encourages lipid accumulation, and diminishes the overall metabolic efficiency of skeletal muscles [10]. If this metabolic imbalance persists, muscle protein synthesis declines, leading to progressive muscle atrophy and exacerbation of the systemic metabolic disturbances associated with T2DM [11].

As T2DM progresses, energy metabolism becomes increasingly dysregulated, primarily due to diminished activity of the AMP-activated protein kinase (AMPK) pathway, a crucial regulator of cellular energy homeostasis [12]. Reduced AMPK activity diminishes glucose uptake, while the downregulation of SIRT1 and PGC-1α impairs mitochondrial biogenesis and function [13,14,15]. Moreover, decreased expression of PPARα and FGF21 contributes to intramuscular lipid accumulation and compromises muscle metabolic efficiency [16,17]. In addition, impaired IRS-1 and AKT signaling in skeletal muscle in T2DM contributes to muscle loss by exacerbating insulin resistance and impairing glucose metabolism [18,19,20]. Furthermore, increased activity of Forkhead box O3 (FOXO3a) enhances the transcription of the Atrogin-1 and Muscle RING-finger protein-1 (MuRF1) coding genes, leading to muscle atrophy and loss of muscle mass [21,22].

Emerging evidence suggests that gut microbiota plays an important role in the development of obesity, insulin resistance, and skeletal muscle dysfunction through the gut–muscle axis [23]. Therefore, characterization of gut microbiota composition may provide additional insights into the mechanisms involved in metabolic regulation and muscle maintenance.

Research on diabetes in murine models has primarily utilized genetic *db*/*db* diabetic mice and mice with chemically induced (streptozotocin [STZ]-treated) diabetes. Currently, *db*/*db* mice constitute a well-established genetic model of T2DM, characterized by dysregulated appetite, obesity, insulin resistance, and hyperglycemia, resulting from a mutation in the leptin receptor [24]. This model is widely employed to investigate the pathophysiological mechanisms of T2DM, which involves both insulin resistance and progressive β-cell dysfunction [25]. In contrast, STZ, a β-cell-selective cytotoxic compound, is commonly administered in conjunction with a high-fat diet (HFD) to induce T2DM in mice that are used as a model [26]. The HFD component promotes insulin resistance, while STZ enters pancreatic β-cells via GLUT2, triggering ATP depletion, inflammation, oxidative stress, and apoptosis, ultimately leading to impaired β-cell function and reduced insulin secretion [27,28]. This combined HFD/STZ-treated model effectively recapitulates the core pathophysiological features of T2DM, including the coexistence of partial β-cell dysfunction and systemic insulin resistance.

Recent studies have demonstrated the efficacy of natural products in the prevention and management of T2DM. *Momordica charantia*, commonly known as bitter melon, is a prominent natural agent used in traditional medicine to treat diabetes. It is rich in bioactive compounds, including charantin, momordicin, and various cucurbitane-type triterpenoids [29,30,31]. These bioactive compounds counteract the pathophysiology of diabetes through a multipronged approach. Their mechanisms of action include a direct reduction in blood glucose levels by improving insulin sensitivity and glucose uptake in peripheral tissues, stimulating insulin secretion from the pancreas, and exerting antioxidant activity to mitigate cellular damage. Additionally, some compounds such as L-arginine enhance vascular function by promoting nitric oxide synthesis, which is often impaired in diabetes [32,33,34].

*Stevia rebaudiana*, a natural sweetener, is increasingly recognized as a bioactive plant with antidiabetic properties that extend beyond its role as a mere sugar substitute, which is also grown in South Korea [35]. Compounds present in stevia, such as stevioside and rebaudioside A, have been reported to possess potential therapeutic effects for the prevention and amelioration of diabetes. These effects encompass the regulation of blood glucose, promotion of insulin secretion, enhancement of insulin sensitivity and antioxidant activity, and maintenance of overall metabolic balance [36,37,38].

In this study, we used EMS, a novel combined extract of *M. charantia* and *S. rebaudiana*, both of which are indigenous domestic plants known for their antidiabetic properties. The natural sweetener *S. rebaudiana* was blended with *M. charantia* to overcome the characteristic bitterness of the latter, which limits its use. It was hypothesized that this formulation would exhibit synergistic antidiabetic effects. To test this hypothesis, we assessed the efficacy of EMS in two mouse models of T2DM, including *db*/*db* mice and STZ-induced diabetic mice. We analyzed diabetes-related markers in mouse blood and examined potential improvements in several metabolic axes of the liver, adipose tissue, and muscle. Additionally, the effects of EMS on insulin resistance, muscle atrophy, sarcopenia, and gut microbiota composition were evaluated.

## 2. Materials and Methods

### 2.1. Materials

EMS samples used in this study were developed and prepared by Hanmi Nutrition, Inc. (Paju, Republic of Korea) and were subsequently provided to the authors for experimental evaluation. Bitter melon extract was selected as the primary functional ingredient for its reported antidiabetic activity, while stevia extract was incorporated as a complementary component due to its potential metabolic benefits and suitability for formulation development. To determine the optimal EMS mixing ratio, a study was conducted in which bitter melon 50% ethanol extract powder and stevia hot water extract powder were combined at ratios of 1:1, 3:1, and 9:1. To evaluate the efficiency of bitterness masking, a sensory evaluation was conducted. A panel scored both sweetness and bitterness intensities using a 5-point structural interval scale (5: extremely strong, 1: non-detectable). Samples of each blending ratio (1:1, 1:2, and 1:9) were evaluated to determine the threshold at which stevia’s natural sweetness effectively diminishes the unpalatable bitterness of the bitter melon extract. The mixtures were screened based on α-glucosidase inhibitory activity, and bitterness-related characteristics were considered only during formulation development. The highest inhibitory activity was observed at a 9:1 ratio; considering the daily safety limits of bitter melon and stevia, the optimal EMS mixing ratio was established as 9:1. An analysis of the indicator components of EMS showed concentrations of 10.58 mg/g stevioside and 9.10 mg/g L-arginine, respectively. During the animal breeding period, the following substances were utilized: metformin (Sigma-Aldrich, St. Louis, MO, USA); STZ (Sigma-Aldrich); D-(+)-Glucose (Sigma-Aldrich); citric acid, trisodium salt dihydrate (Sigma-Aldrich); citric acid (Sigma-Aldrich); and insulin (Gibco, New York, NY, USA). Metformin was used as a positive control substance; STZ was used to induce diabetes; D-(+)-glucose was used for glucose tolerance testing; citric acid and trisodium citrate were used to prepare STZ buffer; and insulin was used for the insulin tolerance test (ITT).

### 2.2. STZ-Induced Diabetic Mouse Model

Sixty male C57BL/6 mice (8-week-old) were obtained from the Central Lab. Animal Inc. (Seoul, Republic of Korea). The sample size was determined to allocate 10 animals per group based on previous studies [39,40]. They were provided with food and water ad libitum. Mice were fed either a 45% high-fat diet (D12451; Research Diet, New Brunswick, NJ, USA) or a 10% fat diet (D12450B; Research Diet) according to their respective experimental groups. After a week of acclimation in a room maintained at 20–25 °C, 50–55% humidity, and a 12 h light/dark cycle, the mice were randomly assigned to six groups. Five groups, excluding the normal control group, were fed an HFD for four weeks. In the fifth week, 50 mg/kg STZ was administered intraperitoneally for three consecutive days. The 50 mg/kg STZ solution was prepared by mixing 0.1 M sodium citrate and 0.1 M citric acid in PBS at a 1:1 ratio, maintaining the pH at 4–4.5; the mixture was sterilized using a 0.22 µm filter and then used to dissolve the STZ powder. To confirm diabetes induction, fasting blood glucose levels (FBGLs) were monitored. Under physiological conditions, fasting blood glucose levels in normal C57BL/6 mice are generally reported to range from approximately 80 to 150 mg/dL. Mice with FBGLs exceeding 250 mg/dL were considered diabetic, consistent with previously established criteria for STZ-induced diabetic mouse models [41,42]. Following confirmation of diabetes induction, the mice were randomly allocated to the respective experimental groups [42]. Mice groups for subsequent oral treatment administration were defined as follows: normal control (CON) group: 10% fat diet + saline; diabetic model (NC) group: STZ + 45% fat diet + saline; positive control (PC) group: STZ + 45% fat diet + 200 mg/kg metformin; EMS low dose (MSL) group: STZ + 45% fat diet + 40 mg/kg EMS; EMS medium dose (MSM) group: STZ + 45% fat diet + 80 mg/kg EMS; and EMS high dose (MSH) group: STZ + 45% fat diet + 120 mg/kg EMS. EMS doses were selected stepwise to assess potential dose-dependent responses. Oral administration was conducted daily at approximately 10:00 AM for six weeks, and body weight, water, and food intake were measured twice weekly. After the administration period, the animals were fasted for 12 h and euthanized by carbon dioxide (CO_2_) inhalation [43]. Blood was collected via cardiac perfusion into EDTA-treated tubes and centrifuged at 3000× *g* for 10 min at 4 °C to separate the serum [44]. Liver, gastrocnemius, epididymal fat, and pancreas tissues were excised, and liver and gastrocnemius tissues were weighed and stored at −80 °C. All animal care and management procedures were approved by the Animal Experiment Ethics Committee of Gachon University (approval no. GU1-2024-IA0062-00) and conducted in compliance with the relevant regulations. A schematic overview of the experimental design and sampling timeline is shown in Supplementary Figure S7.

### 2.3. db/db Mouse Model

Fifty 8-week-old *db*/*db* male mice (BKS.Cg-m+/+Leprdb/+/J[HETERO]) and ten *db*/+ male mice (BKS.Cg-m+/+Leprdb/db/J[DB/DB]) were obtained from the Jackson Laboratory in Yokohama, Kanagawa, Japan. In total, 60 mice were included in the study. After a two-week adaptation period, *db*/*db* mice, but not *db*/+ mice, were fed a HFD containing 45% high-fat (D12451; Research Diet, New Brunswick, NJ, USA) for the first three weeks after adaptation and then a 10% fat diet (D12450B; Research Diet) until the end of the experiment. *db*/+ mice were fed a 10% fat diet throughout the experimental period. Except for the indicated treatments, the rearing conditions and tissue storage methods were consistent with those used for the STZ-treated mouse model group. All animal procedures were approved by the Animal Experiment Ethics Committee of Gachon University (approval no. GU1-2024-IA0032-01) and performed in compliance with the relevant regulations. Mice were divided into the following six groups: nondiabetic control (CON_db) group: *db*/+ mice receiving saline; diabetic control (NC_db) group: *db*/*db* mice receiving saline; positive control (PC_db) group: *db*/*db* mice receiving 200 mg/kg metformin; EMS low dose (MSL_db) group: *db*/*db* mice receiving 40 mg/kg EMS; EMS medium dose (MSM_db) group: *db*/*db* mice receiving 80 mg/kg EMS; and EMS high dose (MSH_db) group: *db*/*db* mice receiving 120 mg/kg EMS (Supplementary Figure S7).

### 2.4. FBGL, Oral Glucose Tolerance Test (OGTT), and Insulin Tolerance Test (ITT)

After a 6 h fasting period with free access to water, FBGL was measured in the experimental animals. FBGL was measured once before oral administration and weekly thereafter for 6 weeks, for a total of 7 measurements. Blood samples were drawn from the mice’s tails, and glucose levels were measured using an Accu-Chek Performa (ACCU-CHEK, Seoul, Republic of Korea). Approximately 2–3 μL of blood was collected from the tail tip at each sampling point, and approximately 2 μL was used for each blood glucose measurement. OGTTs were performed 37 days after the initiation of EMS administration to assess glucose tolerance. Prior to testing, the animals were fasted for 12 h. A baseline blood sample (t = 0) was obtained from the tail vein, after which a glucose solution was administered by oral gavage at a dose of 2 g/kg body weight. Additional blood samples were collected from the tail vein at 15, 30, 60, 90, and 120 min after glucose administration, and blood glucose levels were measured using the same procedure as at baseline. To evaluate systemic insulin sensitivity, an ITT was performed on day 39 of the study. Following a 6 h fast, a baseline blood sample (t = 0) was collected from the tail vein to measure the initial glucose concentration. Subsequently, the animals were administered an intraperitoneal injection of insulin (1.0 U/kg body weight). Additional blood samples were collected from the tail vein at 15, 30, 60, 90, and 120 min post-injection, and blood glucose levels were measured using the same procedure as at baseline. The resulting glucose-time curve was plotted, and the area under the curve (AUC) was calculated to quantify insulin-mediated glucose disposal across the groups.

### 2.5. Measurement of Lipid Levels in Serum

Triglyceride (TG) and total cholesterol (TC) levels (normal ranges are approximately 50–120 mg/dL for TG and 80–140 mg/dL for TC) [45,46] in the mouse serum were measured using TG-S and T-CHO kits (Asanpharm, Hwaseong, Republic of Korea). All measurements were performed in accordance with the manufacturer’s instructions. Biochemical parameters were analyzed to assess the effects of EMS treatment by comparing the diabetic control group with the EMS-treated groups.

### 2.6. Assessment of Hepatocellular Damage and Liver Function Abnormalities

Serum alanine aminotransferase (ALT) and aspartate aminotransferase (AST) levels (normal ranges are approximately 5–30 Karmen/mL for ALT (GPT) and 8–50 Karmen/mL for AST (GOT) [47,48] were measured using Asan set GPT and Asan set GOT assay kits (Asanpharm, Hwaseong, Republic of Korea), respectively. All measurements were performed in accordance with the manufacturer’s instructions. Biochemical parameters were analyzed to assess the effects of EMS treatment by comparing the diabetic control group with the EMS-treated groups.

### 2.7. Enzyme-Linked Immunosorbent Assay (ELISA)

To assess key metabolic hormones, serum samples were analyzed using ELISA. Specific kits were used to quantify adiponectin (Aviscera Bioscience, Santa Clara, CA, USA), leptin (R&D Systems, Minneapolis, MN, USA), and insulin (Millipore, Billerica, MA, USA). Experiments were conducted in accordance with the manufacturer’s instructions, and the final concentrations were determined using a standard curve generated with Gen5 software (version 3.03).

### 2.8. Immunohistochemistry (IHC) Assays

Pancreatic tissues were fixed in 10% neutral-buffered formalin, embedded in paraffin, and sectioned. The sections were subjected to IHC staining using an anti-insulin antibody, and the signals were visualized using a chromogenic substrate. The stained sections were analyzed to assess pancreatic β-cell distribution.

### 2.9. Histological Analysis of Liver and Muscle Tissues

Liver and gastrocnemius tissues were fixed in 10% neutral-buffered formalin, embedded in paraffin, and stained with hematoxylin and eosin (H&E). For muscle tissue, the cross-sectional area (CSA) of individual myofibers was quantified from H&E-stained images using ImageJ software (version 1.5x, National Institutes of Health, Bethesda, MD, USA). To assess hepatic steatosis, frozen liver sections from four mice per group were stained with Oil Red O (ORO), and lipid accumulation in the liver tissues was evaluated. Samples from four randomly selected mice per group were used for histological analysis [49].

### 2.10. Western Blot Analysis

Gastrocnemius tissues (20 mg) were homogenized in 800 μL RIPA buffer containing 8 μL protease and phosphatase inhibitors. The homogenates were incubated on ice for 20 min, then centrifuged at 13,000× *g* for 5 min at 4 °C. The supernatant containing the protein lysate was collected. The protein concentration was quantified using a PRO-MEASURE kit (iNtRON Biotechnology, Seongnam, Republic of Korea) [49,50]. For each sample, 40 µg of protein was resolved by SDS-PAGE and subsequently transferred to a PVDF membrane. The membranes were blocked in 5% skim milk for 1 h at room temperature, followed by an overnight incubation with primary antibodies on a shaker at 4 °C. Western blot analysis was performed to investigate the effects of EMS treatment on energy metabolism, mitochondrial biogenesis, insulin signaling, glucose uptake, and muscle atrophy-related pathways in skeletal muscle. Primary antibodies against p-AMPK (1:1000), AMPK (1:1000), Sirt1 (1:1000), PGC-1α (1:1000), PPARα (1:1000), FGF21 (1:1000), p-IRS-1(Tyr612) (1:500), IRS-1 (1:1000), p-PI3K (1:500), PI3K (1:1000), p-AKT (1:1000), AKT (1:1000), GLUT4 (1:1000), p-FOXO3a (1:1000), FOXO3a (1:1000), Atrogin-1 (1:2000), MuRF1 (1:1000), and GAPDH (1:2000) were purchased from Cell Signaling Technology (Danvers, MA, USA) and Abcam (Cambridge, UK). After incubation, the membranes were washed, incubated with horseradish peroxidase-conjugated secondary antibodies for 1 h, and visualized using ECL solution (iNtRON Biotechnology, Seongnam, Republic of Korea) on a Quant LAS 500 system (GE Healthcare Bio-Sciences AB, Uppsala, Sweden) [51]. Western blot data were normalized to that of GAPDH as the housekeeping control. Band intensities were quantified using the system’s analysis software with background correction. Target protein levels were normalized to those of GAPDH as an internal control. All experiments were performed in triplicate, with six mice per group.

### 2.11. Statistical Analysis

Data were analyzed using GraphPad Prism 10.3.1 (GraphPad Software Inc., San Diego, CA, USA), and results are presented as mean ± SD. Normality and homogeneity of variance were assessed using the Shapiro–Wilk and Brown–Forsythe tests, respectively, prior to statistical analyses. For all in vivo experiments, one-way analysis of variance (ANOVA) followed by Dunnett’s multiple comparisons test was used to test differences between the groups; statistical significance was set at *p* < 0.05. * *p* < 0.05, ** *p* < 0.01, *** *p* < 0.001, and **** *p* < 0.0001.

### 2.12. Gut Microbiome Sequence Analysis

Amplicon sequences obtained using a next-generation sequencing platform were imported into the Quantitative Insights into Microbial Ecology (QIIME) version 2 pipeline via the pair-end read import module [52]. These paired-end reads were subjected to quality control analysis (QCA) using the divisive amplicon denoising algorithm 2 (DADA2) [53]. After manual visualization, denoising, trimming, and removal of low-quality reads and chimeras were performed using QCA. The amplicon sequence variants (ASVs) obtained after the QCA were subjected to phylogenetic analysis using multiple sequence alignments (MSA) through FastTree version 2.1.10 [54,55].

### 2.13. Taxonomic Annotation of ASVs

The taxonomic annotation of all ASVs was performed using the Greengenes 13_8 99% OTU-based taxonomy classifier in the QIIME 2 pipeline, which uses the naïve Bayes algorithm for taxonomic classification [56]. The graphical representation of the taxonomic annotation of all samples was drawn using the taxabar module of the QIIME 2 pipeline (FastTree version 2.1.10) [57].

### 2.14. Alpha- and Beta-Diversity Analysis

Diversity analysis of the samples was performed using the QIIME 2 pipeline. Alpha diversity and alpha diversity evenness were studied using Faith’s phylogenetic diversity and Pielou’s evenness, respectively. Beta diversity was assessed using qualitative and quantitative community dissimilarity metrics. Jaccard and Bray–Curtis distance measures were used in the beta diversity analysis. 

### 2.15. Taxa Abundance per Group

The differential abundance of taxa among groups was also examined, as in our previous studies [58,59,60,61,62]. The linear discriminant analysis effect size (LEfSe) program was used to identify differentially abundant taxa using a Docker-based Implementation. The ASV table, metadata, and species-level collapsed taxonomy results were used for differential abundance analysis. Graphical representations of differentially abundant taxa across groups were generated as bar graphs and cladograms [58].

## 3. Results

### 3.1. Effects of EMS on FBGL, OGTT, and ITT

**Figure 1:**
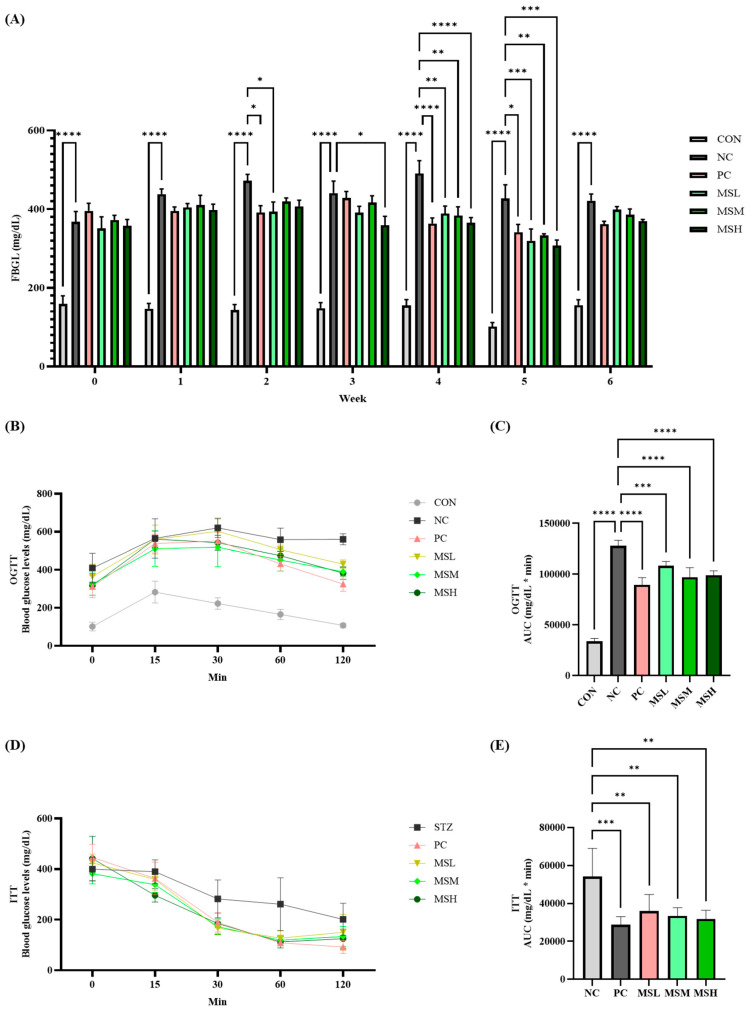
Effects of a novel combined extract of *M. charantia* and *S. rebaudiana* (EMS) on glucose and insulin tolerance in a streptozotocin (STZ)-induced diabetes mouse model. (**A**) FBGL, (**B**) OGTT levels, (**C**) AUC of OGTT, (**D**) ITT levels, and (**E**) AUC of ITT of STZ-induced diabetic mice. The data are presented as the mean ± SD. * *p* < 0.05, ** *p* < 0.01, *** *p* < 0.001, and **** *p* < 0.0001 vs. the NC (diabetic model) group.

Following the confirmation of STZ-induced diabetes at week 0, EMS treatment led to a time-dependent reduction in FBGL. Blood analyses were performed immediately after diabetes confirmation, and elevated blood glucose levels were observed in all groups except the CON group. However, by week 2, significant reductions in FBGL were observed in the MSL and MSH groups compared with the NC group, and these reductions were evident in all EMS-treated groups at weeks 4 and 5. Although FBGL remained lower in all EMS-treated groups at week 6 than in the NC group, the difference was not statistically significant (Figure 1A).

An OGTT performed on day 37 confirmed marked improvements in glucose handling, and all EMS-treated groups exhibited significantly higher glucose clearance and lower AUC than the NC group (Figure 1B,C). To further assess insulin sensitivity, the ITT was conducted on day 39. EMS-treated mice showed a significant decrease in blood glucose levels after insulin administration compared with those from the NC group. Quantitative analysis of the AUC confirmed that all the EMS-treated groups exhibited significantly improved insulin tolerance (Figure 1D,E).

### 3.2. Effects of EMS Treatment on Liver Enzymes, Metabolic Hormones, and Lipid Profiles

**Figure 2:**
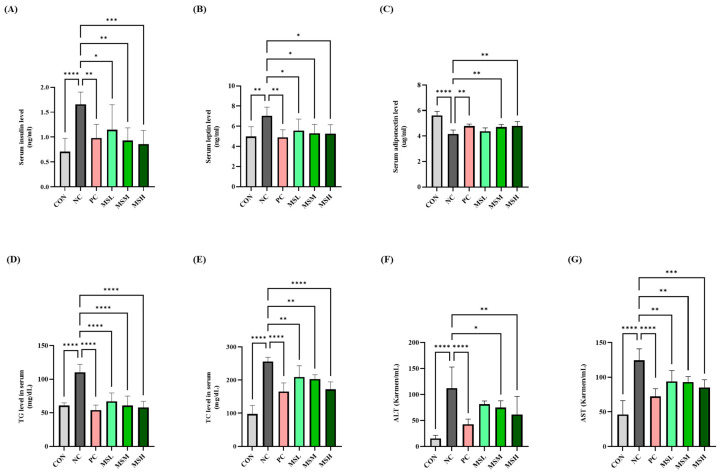
Effects of a novel combined extract of *M. charantia* and *S. rebaudiana*(EMS) on liver enzymes, metabolic hormones, and lipid profiles in a streptozotocin (STZ)-induced diabetes mouse model. (**A**) Insulin, (**B**) leptin, (**C**) adiponectin, (**D**) TG, (**E**) TC, (**F**) ALT, and (**G**) AST levels in STZ-induced diabetic mice. The data are presented as the mean ± SD. * *p* < 0.05, ** *p* < 0.01, *** *p* < 0.001, and **** *p* < 0.0001 vs. the NC (diabetic model) group.

To evaluate the effects of EMS on metabolic regulation, serum levels of insulin, leptin, and adiponectin were measured in STZ-induced diabetic mice (Figure 2A–C). While the NC group exhibited metabolic dysregulation, the PC- and EMS-treated groups showed significant improvements in metabolic markers, including reductions in insulin and leptin levels. In addition, TG and TC levels were elevated in the NC group but were significantly decreased in the PC- and EMS-treated groups, indicating improved serum lipid profiles (Figure 2D,E).

Similar effects were observed in *db*/*db* mice (Supplementary Figure S1A–E). The NC_db group exhibited a hormonal imbalance characterized by elevated leptin and reduced adiponectin levels. In contrast, the PC_db- and EMS_db-treated groups showed alleviation in these imbalances, exhibiting decreased leptin and increased adiponectin levels. Furthermore, both the TG and TC levels were reduced in these groups, supporting the lipid-lowering effects of the treatments.

To assess hepatocellular damage, serum ALT and AST levels were measured (Figure 2F). The NC group showed significantly elevated ALT and AST levels compared with the CON group, whereas the PC- and EMS-treated groups exhibited lower levels, indicating attenuation of liver injury.

### 3.3. Protective Effects of EMS on Pancreatic β-Cell Distribution, Hepatic Histopathological Damage, and Lipid Accumulation in STZ-Induced Diabetic Mice

**Figure 3:**
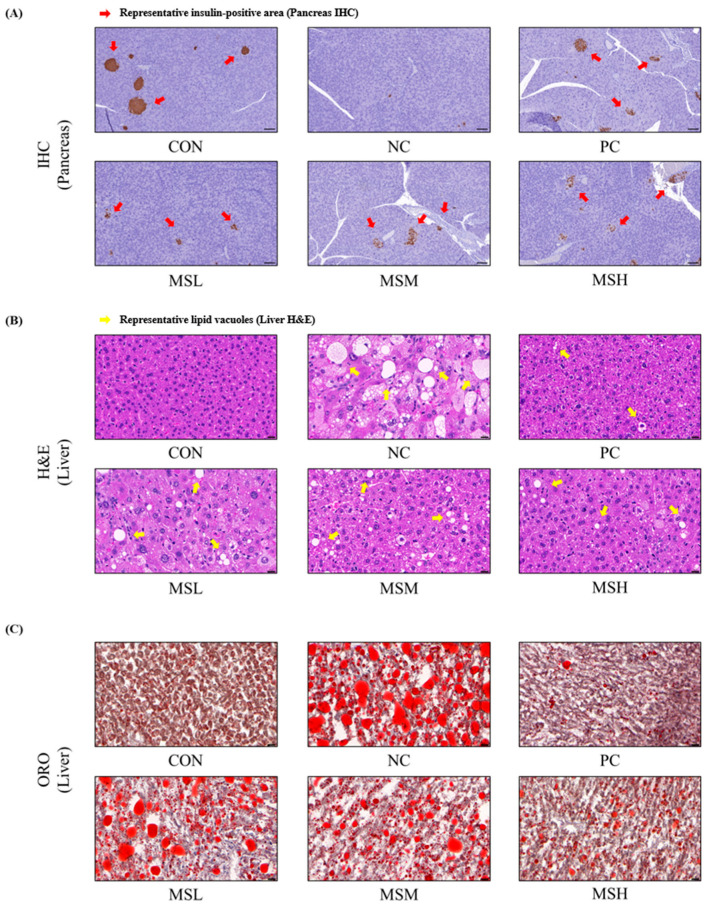
Protective effects of a novel combined extract of *M. charantia* and *S. rebaudiana* (EMS) on pancreatic β-cell preservation, hepatic histopathological damage, and lipid accumulation in streptozotocin (STZ)-induced diabetic mice. (**A**) IHC staining of pancreas sections and (**B**) H&E and (**C**) ORO staining of liver sections from STZ-induced diabetic mice. Scale bars: 100, 20, and 20 μm, respectively.

Pancreatic β-cell alterations were evaluated by IHC staining (Figure 3A). The PC- and EMS-treated groups showed a greater number of β-cells than the NC group, suggesting that EMS administration may contribute to the maintenance of pancreatic β-cell distribution. Histological analysis of liver tissues using H&E staining revealed increased lipid accumulation and irregular hepatocellular morphology in the NC group compared to those in the CON group. In contrast, the PC group showed attenuated histopathological abnormalities. Notably, the EMS-treated groups demonstrated dose-dependent improvements, including reduced lipid accumulation and restoration of hepatic architecture, indicating that EMS effectively alleviated liver damage (Figure 3B). Consistent with these findings, ORO staining revealed marked hepatic lipid accumulation in the NC group, which was reduced in the PC group. All EMS-treated groups exhibited a dose-dependent reduction in hepatic lipid droplet numbers (Figure 3C).

### 3.4. Effects of EMS on the Improvement of Muscle Fibers

**Figure 4:**
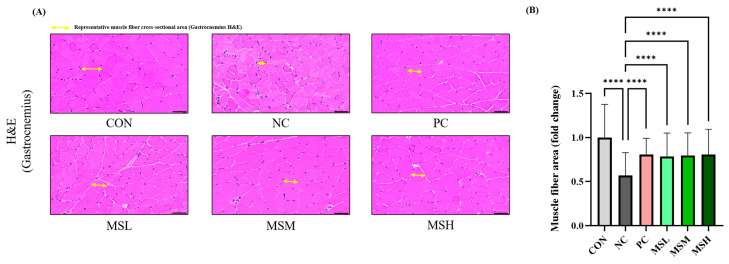
A novel combined extract of *M. charantia* and *S. rebaudiana* (EMS) treatment leads to increased muscle fiber size in the streptozotocin (STZ)-induced diabetes mouse model. (**A**) H&E staining of gastrocnemius muscles from STZ-induced diabetic mice. Scale bar: 50 μm. (**B**) Muscle fiber size of STZ-induced diabetic mice. The data are presented as the mean ± SD. **** *p* < 0.0001 vs. the NC (diabetic model) group.

Muscle fiber morphology was evaluated by H&E staining of gastrocnemius muscle tissue samples (Figure 4A). The NC group exhibited a significant reduction in muscle fiber cross-sectional area compared with that in the CON group, whereas the PC and all EMS-treated groups showed significant increases in fiber size relative to that in the NC group (Figure 4B).

### 3.5. Effects of EMS on Markers of Lipid Metabolism, Mitochondrial Function, and Insulin Signaling in Gastrocnemius Muscle

**Figure 5:**
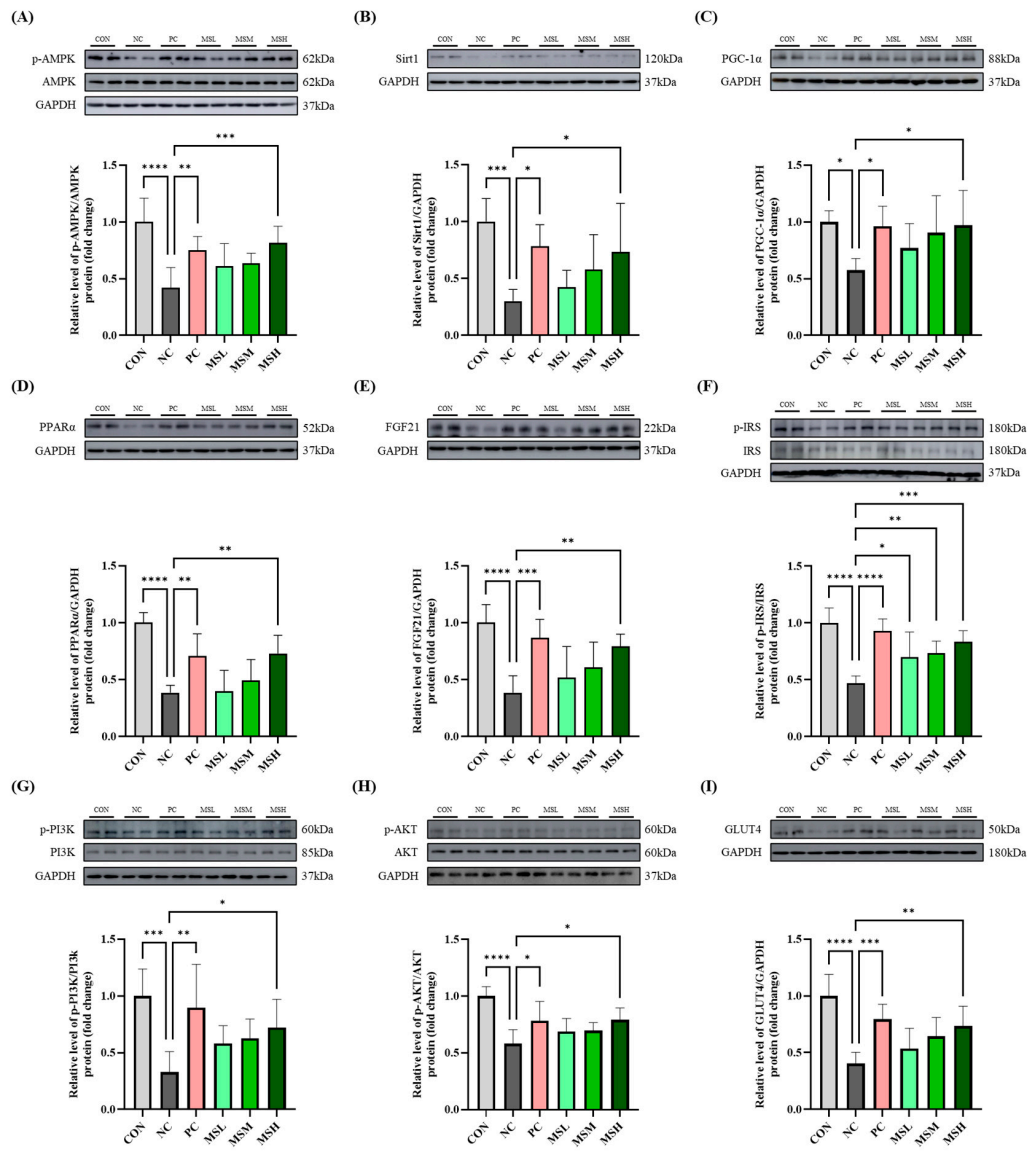
Effects of a novel combined extract of *M. charantia* and *S. rebaudiana* (EMS) on lipid metabolism, mitochondrial function, and insulin signaling in skeletal muscle. (**A**) AMPK, (**B**) Sirt1, (**C**) PGC-1α, (**D**) PPARα, (**E**) FGF21, (**F**) IRS, (G) PI3K, (**H**) AKT, and (**I**) GLUT4 protein levels in the gastrocnemius of streptozotocin (STZ)-induced diabetic mice. GAPDH was used as an internal control. The data are presented as the mean ± SD. * *p* < 0.05, ** *p* < 0.01, *** *p* < 0.001, and **** *p* < 0.0001 vs. the NC (diabetic model) group.

To assess skeletal muscle metabolism in STZ-induced diabetic mice, the levels of key regulatory proteins in the gastrocnemius muscle were analyzed (Figure 5A–E). All marker levels were significantly lower in the NC group than in the CON group, whereas those in the PC group were significantly higher. EMS treatment effectively reversed these impairments, with all the EMS-treated groups exhibiting elevated marker expression levels. Notably, the MSH group showed significant restoration of marker levels. These findings indicate that EMS enhances mitochondrial function and metabolic pathways in skeletal muscle.

Similar trends were observed in *db*/*db* mice (Supplementary Figure S2A–D). The NC_db group showed reduced expression of AMPK, Sirt1, PGC-1α, and PPARα, and EMS treatment restored these marker levels. Among the groups, the MSH_db group showed a significant recovery, confirming the metabolic benefits of EMS observed in the murine model of STZ-induced diabetes.

To further evaluate muscle insulin signaling, IRS-1, PI3K, AKT, and GLUT4 expression levels were analyzed in STZ-induced diabetic mice (Figure 5F–I). The NC group exhibited impaired insulin signaling, as evidenced by the reduced expression of all markers. EMS treatment restored these markers, and a significant increase was observed in the MSH group.

### 3.6. Effects of EMS on the FOXO3a/Atrogin-1/MuRF1 Muscle Atrophy Pathway

**Figure 6:**
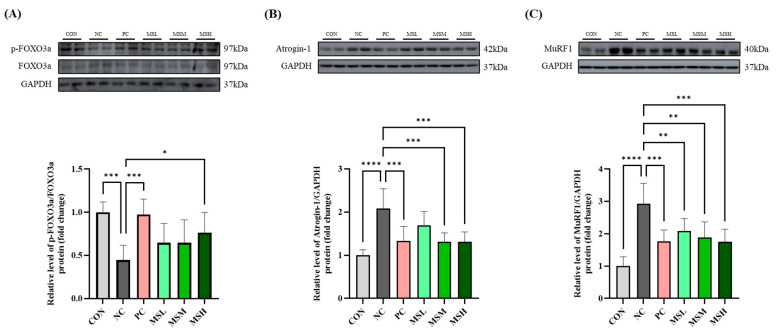
Effects of a novel combined extract of *M. charantia* and *S. rebaudiana* (EMS) on improving the expression of proteins associated with muscle atrophy and sarcopenia in the gastrocnemius muscle of streptozotocin (STZ)-induced diabetic mice. (**A**) FOXO3a, (**B**) Atrogin-1, and (**C**) MuRF1 protein levels in the gastrocnemius muscle of STZ-induced diabetic mice. GAPDH was used as an internal control. The data are presented as the mean ± SD. * *p* < 0.05, ** *p* < 0.01, *** *p* < 0.001, and **** *p* < 0.0001 vs. the NC (diabetic model) group.

To determine whether EMS mitigated muscle atrophy in STZ-induced diabetic mice, the expression of key proteins involved in the muscle degradation pathway was analyzed (Figure 6A–C). The NC group exhibited a pro-atrophic profile characterized by activation of the FOXO3a/Atrogin-1/MuRF1 axis, as evidenced by reduced inhibitory phosphorylation of FOXO3a and increased expression of Atrogin-1 and MuRF1. Notably, EMS treatment suppressed this pathway. In EMS-treated groups, MuRF1 expression was significantly reduced at all doses, whereas Atrogin-1 expression was reduced at medium and high doses. Notably, high-dose EMS increased inhibitory phosphorylation of FOXO3a, thereby suppressing Atrogin-1 activation.

Similar patterns were observed in the *db*/*db* mouse model (Supplementary Figure S3A–C), in which EMS treatment increased FOXO3a phosphorylation and reduced Atrogin-1 and MuRF1 expression.

### 3.7. Microbiome and Diversity Analysis in STZ-Induced Diabetic Mice

In total, 2,355,572 paired-end reads from 14 samples were analyzed. After QCA (preprocessing), 18,995 unique features/ASVs/OTUs were identified in all four groups.

The alpha diversity and alpha diversity evenness (Pielou’s evenness) were slightly (nonsignificantly) increased in the treatment groups compared to those in the CON group, and significant differences were only observed between the CON and NC groups (Supplementary Figure S4A,B).

A slight but significant increase in beta diversity was observed in the treatment groups compared to that in the CON group (Supplementary Figure S5A). A 3D beta diversity plot based on the Jaccard distance revealed that the treatment group samples were skewed to the left, whereas the control group samples were scattered to the right (Supplementary Figure S5B).

### 3.8. Taxonomy Annotation and Differential Abundance of Taxa in Microbiome Sample Sequences

**Figure 7:**
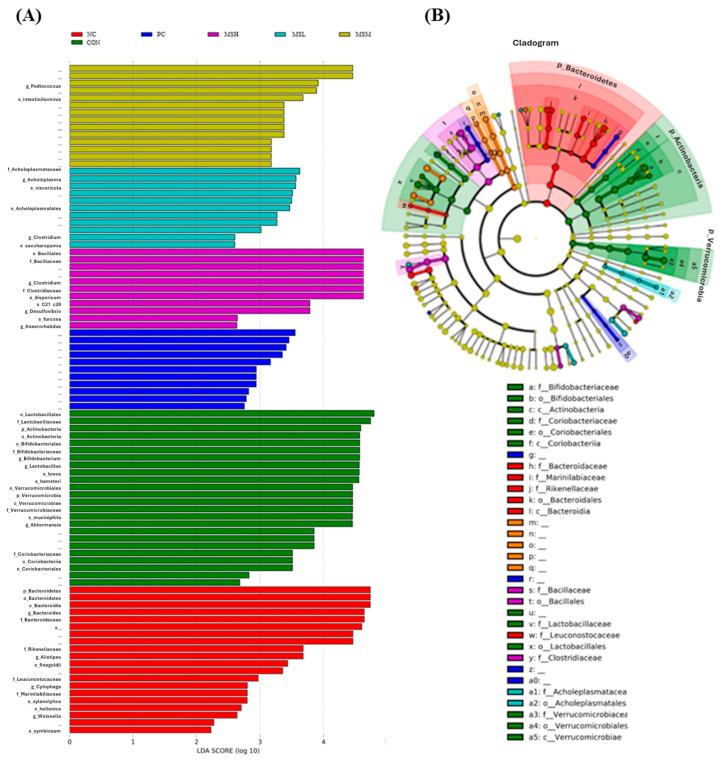
Differential abundance of microbial species in treated and untreated streptozotocin (STZ)-induced diabetic mice. (**A**) Histogram of LDA scores and (**B**) cladogram for differentially abundant bacterial taxa in the microbiome of mice from different testing groups. The cladogram was calculated using LEfSe and displayed according to effect size. Only those taxa with an LDA significant threshold of 2 are shown.

Taxonomic annotation of all sample sequences revealed that the most abundant phylum in nearly all the samples was Firmicutes, followed by Actinobacteria and Bacteroidetes (Supplementary Figure S6). A species-level differential abundance analysis revealed that *Barnesiella intestinihominis*, *B. viscericola*, *Clostridium saccharogumia*, *C. disporicum*, *Desulfovibrio C21_c20*, and *Anaerorhabdus furcosa* were the most abundant species in the treatment groups (Figure 7). Similarly, *Bifidobacterium breve*, *Lactobacillus hamsteri*, and *Akkermansia muciniphila* were the most abundant species in the PC group (Figure 7). Among these species, *B. intestinihominis* [63] from the MSL group, as well as B. breve [64] and *Akkermansia muciniphila* [65] from the PC group, have been reported to have positive effects on diabetes. 

## 4. Discussion

Although various pharmacological interventions have been developed to treat T2DM, they are often accompanied by undesirable side effects. Consequently, interest in natural antidiabetic functional foods that provide therapeutic benefits with minimal adverse effects is growing [64]. Among the numerous natural compounds investigated, stevia and bitter melon have garnered particular attention because of their potent antidiabetic properties [35,65]. The main bioactive components of EMS, the formulation combining bitter melon and stevia extracts used in this study, are stevioside and L-arginine. Stevioside and L-arginine were used as quality-control markers; however, further phytochemical characterization and batch standardization are required to improve reproducibility. Following oral administration, stevioside is metabolized by gut microbiota and the liver into active metabolites that exert glucose-lowering and metabolic effects [66]. L-arginine is absorbed in the intestine via cationic amino acid transporters (CATs) and distributed systemically through the bloodstream [67]. Once absorbed, stevioside and L-arginine activate AMPK, thereby exerting positive effects on metabolic regulation. This activation promotes protein synthesis, enhances antioxidant defense mechanisms, and improves the overall metabolic function [68,69]. Accordingly, the present study investigated whether EMS administration enhanced the antidiabetic function of metabolic tissues in STZ-induced and *db*/*db* diabetic mice.

The administration of EMS effectively suppressed the elevation of FGBL levels in STZ-induced diabetic mice, suggesting that EMS ameliorated hyperglycemia in diabetes. Consistent with these findings, IHC staining of pancreatic tissues revealed restoration of insulin secretion function, indicating that the glucose-lowering effect of EMS may be associated with improved pancreatic β-cell activity. Moreover, reductions in ALT and AST levels, together with histological observations from H&E-stained liver sections, demonstrated that EMS mitigated hepatocellular injury and improved hepatic architecture. In both STZ-induced and *db*/*db* mice, EMS treatment significantly decreased TG and TC levels compared to those in the respective NC and NC_db groups. Consistently, ORO staining of hepatic tissues revealed a marked reduction in lipid deposition in EMS-treated STZ-induced diabetic mice. ORO staining is a useful method for visualizing lipid accumulation; however, its limitations in quantification necessitate additional hepatic lipid analysis and blinded histopathological scoring for a more comprehensive assessment. Collectively, these findings indicate that EMS exerts protective effects against the liver damage and lipid accumulation associated with T2DM.

T2DM is characterized by insulin resistance, reduced fatty acid oxidation, and impaired glucose uptake. These metabolic abnormalities are associated with altered circulating levels of insulin, leptin, and adiponectin [70,71]. In T2DM, insulin resistance triggers compensatory hypersecretion of insulin from pancreatic β-cells, persistently elevated leptin levels have been implicated in amplifying systemic inflammation and worsening insulin resistance, thereby promoting disease progression [72,73]. Additionally, adipocyte hypertrophy and inflammation contribute to reduced adiponectin secretion [74]. In this study, EMS treatments decreased circulating insulin and leptin levels and increased adiponectin levels in diabetic mice, thereby ameliorating the secretory abnormalities of such markers. These findings suggest that EMS exerts antidiabetic effects by improving glycemic regulation, enhancing fatty acid oxidation, and modulating appetite control.

Muscle atrophy and sarcopenia are common complications associated with T2DM [75]. In the present study, the NC group exhibited a marked reduction in muscle fiber cross-sectional area, indicative of muscle atrophy. Notably, high-dose EMS administration significantly restored muscle fiber size, suggesting attenuation of muscle fiber atrophy under diabetic conditions.

The AMPK/Sirt1/PGC-1α axis is a key pathway associated with energy metabolism and mitochondrial function. This axis supports mitochondrial function and muscle preservation in skeletal muscle. In the context of T2DM, the inhibition of this signaling pathway exacerbates fat accumulation, inflammation, and muscle atrophy [76,77]. In skeletal muscle, PPARα regulates fatty acid oxidation and lipid homeostasis, while FGF21 activates AMPK and enhances mitochondrial energy metabolism [78,79]. In this study, the analysis of protein expression in the gastrocnemius muscle of STZ-induced and *db*/*db* diabetic mice revealed that EMS administration significantly increased the expression of these metabolic regulators compared with that in untreated diabetic controls. Collectively, these findings suggest that EMS is associated with improved muscle metabolism and mitochondrial function in T2DM.

The IRS-1/PI3K/AKT signaling axis plays a crucial role in regulating glucose metabolism and protein synthesis in the skeletal muscle through insulin signaling pathways. Upon insulin receptor activation, IRS-1 undergoes phosphorylation, activating the PI3K/AKT cascade, which promotes glucose uptake, suppresses hepatic gluconeogenesis, and maintains muscle insulin sensitivity [80]. In T2DM, this signaling axis is disrupted, leading to insulin resistance, reduced glucose utilization, and impaired muscle protein synthesis [81]. In the present study, EMS administration restored the suppressed expression of IRS-1 and AKT, suggesting that EMS may improve insulin sensitivity in skeletal muscle under diabetic conditions. The observed increase in GLUT4 protein expression suggests an enhanced capacity for glucose transport. Furthermore, the possibility that GLUT4 translocation to the cell membrane is facilitated by AKT signaling cannot be excluded [82]. Further studies employing AMPK and SIRT1 inhibition, cell-based mechanistic validation, and assessment of GLUT4 membrane translocation are warranted to clarify the molecular mechanisms underlying the antidiabetic effects of EMS.

**Figure 8:**
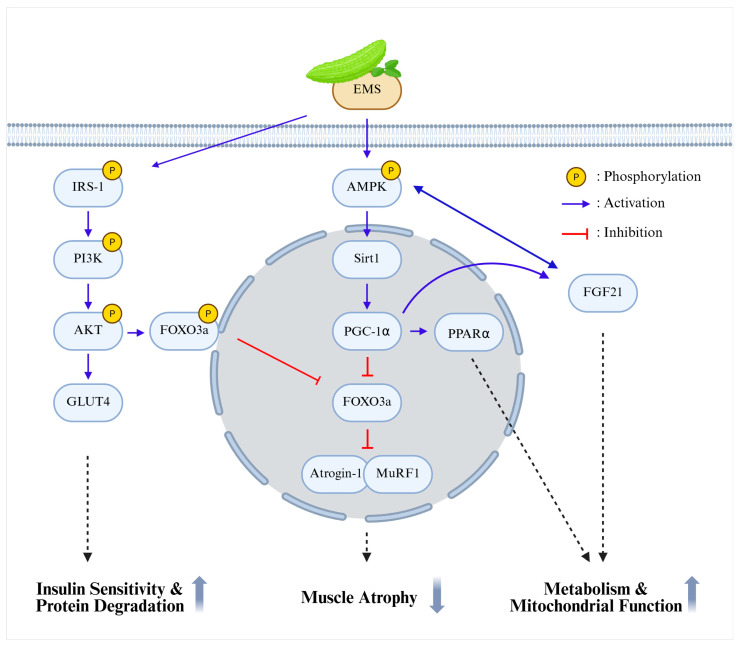
Graphical pathway diagram showing the effects of EMS, a novel extract combining *Momordica charantia* and *Stevia rebaudiana*, on muscle dysfunction. The Figure 8 in this manuscript was Created in BioRender. Lee, H. (2026). https://BioRender.com/82ppue6 (accessed on 21 May 2026).

AKT and PGC-1α negatively regulate the transcription factor FOXO3a via inhibitory phosphorylation [83]. FOXO3a becomes hyperactive when inhibitory signals from pathways such as AKT and PGC-1α are reduced, leading to increased expression of Atrogin-1 and MuRF1, which promote protein degradation [84,85]. Therefore, suppression of this degradation pathway may contribute to the protective effects of EMS against muscle loss (Figure 8).

Taxonomic annotation results indicated a generally consistent bacterial composition at the phylum level; however, species-level differential abundance analysis revealed that specific microorganisms, including *B. intestinihominis*, *B. viscericola*, *C. saccharogumia*, *C. disporicum*, and *A. furcosa*, were commonly abundant in the treatment groups. These findings suggest that, while changes in the overall diversity of the gut microbial community were limited across treatments, selective regulation of specific microbial groups occurred. Furthermore, as these microorganisms have been reported to be associated with gut immune regulation, short-chain fatty acid production, and metabolic pathways, the observed alterations in microbial composition may be related to changes in the gut microbial environment following EMS treatment [86,87]. However, caution is warranted in this interpretation as some strains may exhibit conflicting effects depending on the gut environment. Notably, *B. intestinihominis*, whose abundance increased in the MSL group, has been reported to be associated with the maintenance of intestinal immune homeostasis and the suppression of pathogens, suggesting a potential association between these bacterial changes and intestinal or metabolic health [63,88].

Furthermore, the identified taxa are relevant to T2DM, as alterations in specific gut microbial populations have been associated with insulin sensitivity, inflammation, and metabolic dysfunction. Therefore, the observed taxa may provide additional insight into the potential association between EMS treatment and metabolic regulation through modulation of the gut microbiota [88,89].

Based on body surface area conversion, the EMS doses used in this study correspond to an estimated daily intake of approximately 227–681 mg for a 70 kg adult, although further studies are needed to establish the optimal dosage, evaluate long-term safety, and assess potential herb–drug interactions before clinical application. Although long-term safety studies and human clinical trials are still needed, the present findings suggest that EMS may be a promising functional food for managing T2DM and its associated complications.

In summary, this study demonstrated that EMS is a multi-target functional ingredient for the treatment of T2DM. It effectively regulates blood glucose levels, mitigates lipid accumulation, improves insulin sensitivity, and enhances mitochondrial function in muscle tissues. In addition to its metabolic effects, EMS was associated with attenuation of muscle fiber atrophy, a characteristic feature of T2DM. Future studies incorporating functional assessments will be valuable for further establishing the physiological relevance of the observed molecular and histological changes.

## Data Availability

The original contributions presented in this study are included in the article/Supplementary Materials. Further inquiries can be directed to the corresponding authors.

## Supplementary Materials

The following supporting information can be downloaded at: https://www.mdpi.com/article/10.3390/foods15132364/s1, Figure S1: Effects of EMS on liver enzymes, metabolic hormones, and lipid profiles in *db*/*db* mice. (A) Insulin, (B) leptin, (C) adiponectin, (D) TG, and (E) TC levels of *db/db* mice from different treatment groups. The data are presented as the mean ± SD. * *p* < 0.05, ** *p* < 0.01, *** *p* < 0.001, and **** *p* < 0.0001 vs. the NC_db group. Figure S2: Effects of EMS on proteins related to diabetic metabolic and mitochondrial function in the gastrocnemius muscle of *db*/*db* mice. (A) AMPK, (B) Sirt1, (C) PGC-1α, and (D) PPARα protein levels in the gastrocnemius of *db/db* mice from different treatment groups. GAPDH was used as the internal control. The data are presented as the mean ± SD. * *p* < 0.05, ** *p* < 0.01, *** *p* < 0.001, and **** *p* < 0.0001 vs. the NC_db group. Figure S3: Effects of EMS on improving the expression of proteins associated with muscle atrophy and sarcopenia in the gastrocnemius muscle of mice of a *db*/*db* diabetes model. (A) FOXO3a, (B) Atrogin-1, and (C) MuRF1 protein levels in the gastrocnemius of *db/db* mice from different treatment groups. GAPDH was used as the internal control. The data are presented as the mean ± SD. * *p* < 0.05, ** *p* < 0.01, *** *p* < 0.001, and **** *p* < 0.0001 vs. the NC_db group. Figure S4: Plots showing alpha diversity and evenness across the microbiomes of STZ-induced diabetic mice from different treatment groups. (A) Alpha diversity and (B) alpha diversity evenness plot. Figure S5: Plots showing beta diversities across the microbiomes of STZ-induced diabetic mice from different treatment groups. (A) Beta diversity group significance box plot and (B) beta diversity 3D plot. Figure S6: Bar plots depicting taxonomical annotation (at the phylum level) of the microbiomes of STZ-induced diabetic mice from all six treatment groups. Figure S7: Experimental design and sampling timeline in this study.

## Abbreviations

The following abbreviations are used in this manuscript:

T2DM	Type 2 diabetes mellitus
STZ	Streptozotocin
AMPK	AMP-activated protein kinase
FOXO3a	Forkhead box O3
HFD	High-fat diet
EMS	a novel combined extract of *M. charantia* and *S. rebaudiana*
CON	Normal control
NC	Diabetic model
PC	Positive control
MSL	EMS low dose
MSM	EMS medium dose
MSH	EMS high dose
CON_db	Normal control (*db*/*+*)
NC_db	Diabetic model (*db*/*db*)
PC_db	Positive control (*db*/*db*)
MSL_db	EMS low dose (*db*/*db*)
MSM_db	EMS medium dose (*db*/*db*)
MSH_db	EMS high dose (*db*/*db*)
AUC	Area under the curve
TG	Triglyceride
TC	Total cholesterol
ALT	Alanine aminotransferase
AST	Aspartate aminotransferase
ELISA	Enzyme-linked immunosorbent assay
IHC	Immunohistochemistry
H&E	Hematoxylin and eosin
ORO	Oil Red O
QIIME	Quantitative Insights into Microbial Ecology
QCA	Quality control analysis
DADA2	Divisive amplicon denoising algorithm 2
ASVs	Amplicon sequence variants
MSA	multiple sequence alignments
LEfSe	Linear discriminant analysis effect size
