# Supplementary material for: A Novel Blend of Momordica charantia and Stevia rebaudiana Extracts Ameliorates Metabolic Dysfunction and Muscle Atrophy in Type 2 Diabetic Mice
Source: Foods. 2026 Jul 3;15(13):2364. doi: 10.3390/foods15132364 (PMC13362076; doi:10.3390/foods15132364)
Supplement: Supplementary file 1 [file foods-15-02364-s001.zip › foods-4345267-supplementary.pdf]

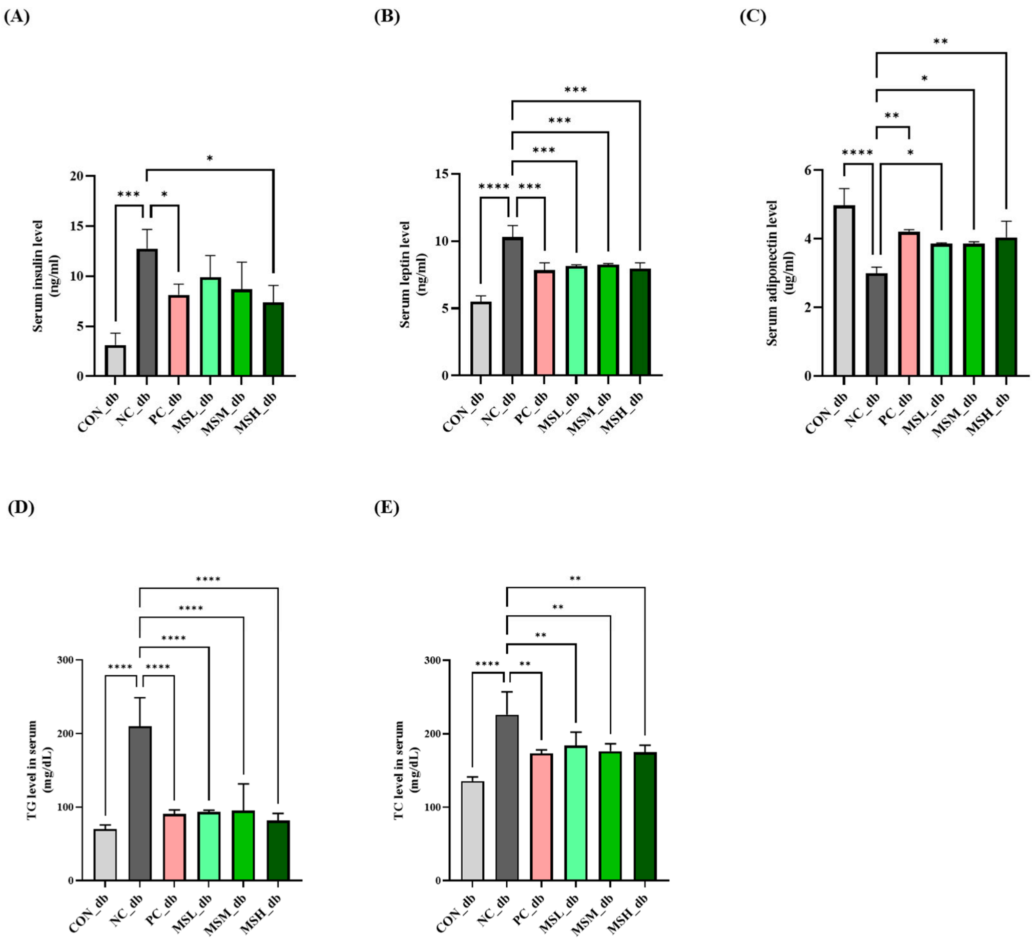


**Supplementary Figure S1.** Effects of EMS on liver enzymes, metabolic hormones, and lipid profiles in *db/db* mice. (A) Insulin, (B) leptin, (C) adiponectin, (D) TG, and (E) TC levels of *db/db* mice from different treatment groups. The data are presented as the mean ± SD. * $p < 0.05$, ** $p < 0.01$, *** $p < 0.001$, and **** $p < 0.0001$ vs. the NC_db group.

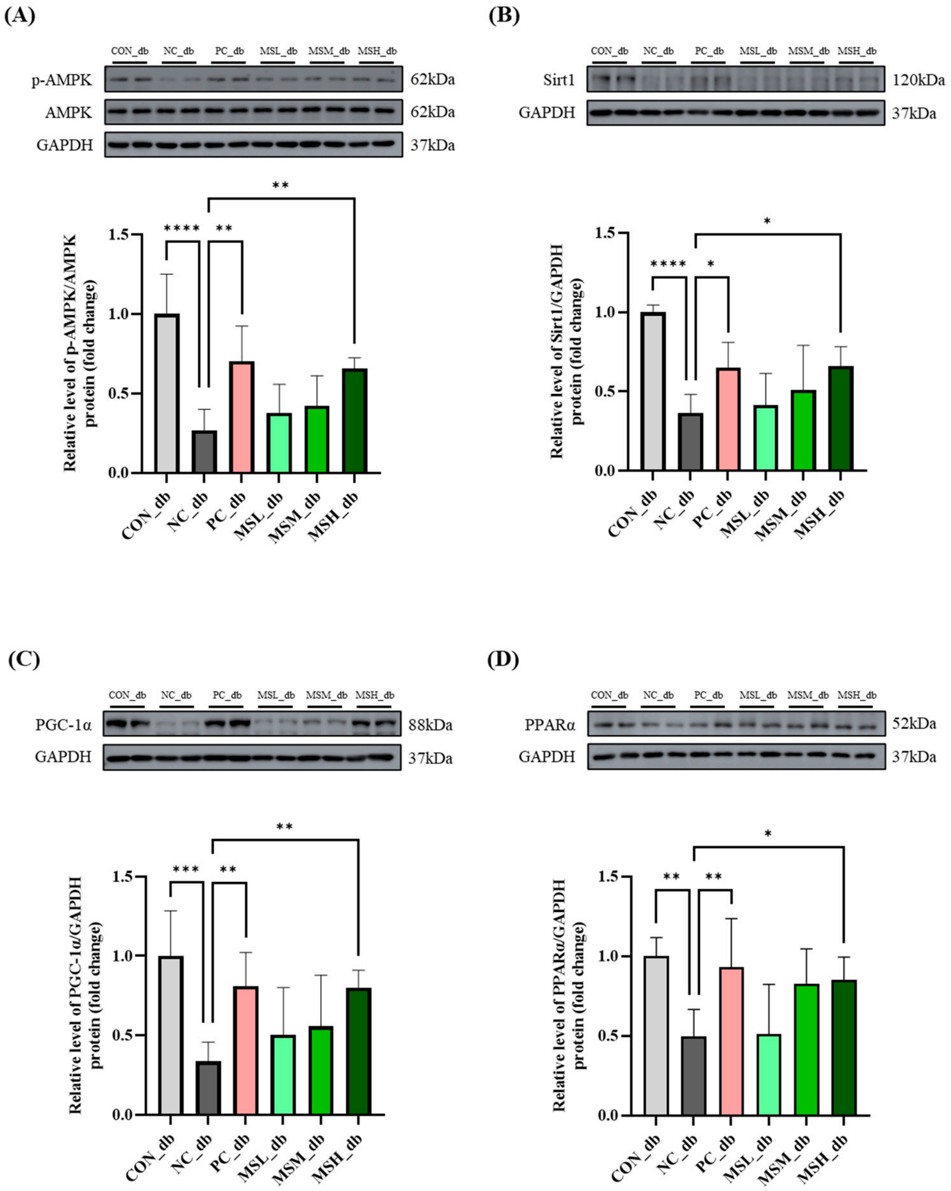


**Supplementary Figure S2.** Effects of EMS on proteins related to diabetic metabolic and mitochondrial function in the gastrocnemius muscle of *db/db* mice. (A) AMPK, (B) Sirt1, (C) PGC-1$\alpha$, and (D) PPAR$\alpha$ protein levels in the gastrocnemius of *db/db* mice from different treatment groups. GAPDH was used as the internal control. The data are presented as the mean ± SD. * $p < 0.05$, ** $p < 0.01$, *** $p < 0.001$, and **** $p < 0.0001$ vs. the NC_db group.

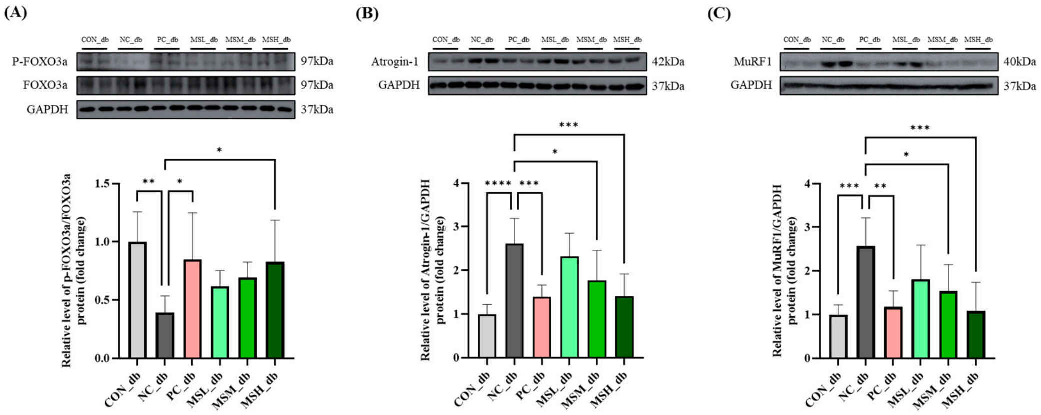


**Supplementary Figure S3.** Effects of EMS on improving the expression of proteins associated with muscle atrophy and sarcopenia in the gastrocnemius muscle of mice of a *db/db* diabetes model**.** (A) FOXO3a, (B) Atrogin-1, and (C) MuRF1 protein levels in the gastrocnemius of *db/db* mice from different treatment groups. GAPDH was used as the internal control. The data are presented as the mean ± SD. * $p < 0.05$, ** $p < 0.01$, *** $p < 0.001$, and **** $p < 0.0001$ vs. the NC_db group.

(A)

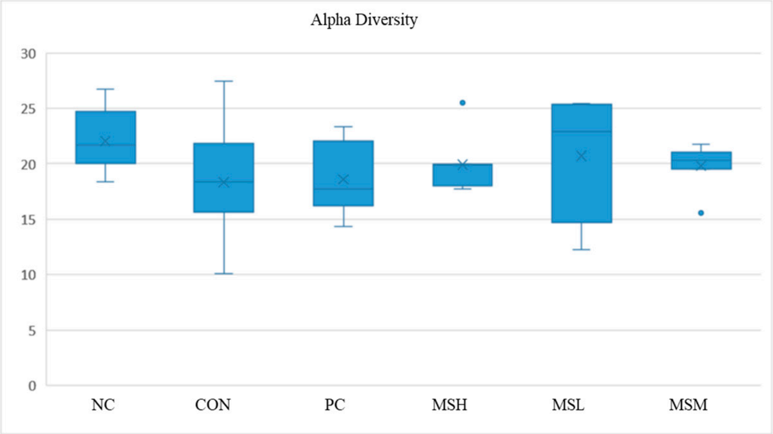


(B)

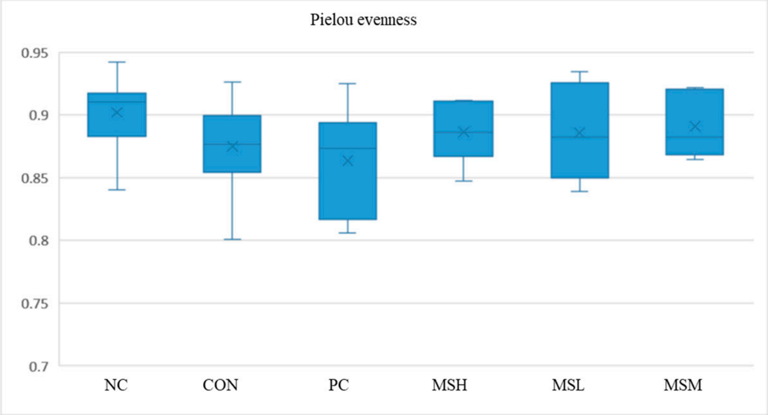


**Supplementary Figure S4.** Plots showing alpha diversity and evenness across the microbiomes of STZ-induced diabetic mice from different treatment groups. (A) Alpha diversity and (B) alpha diversity evenness plot.

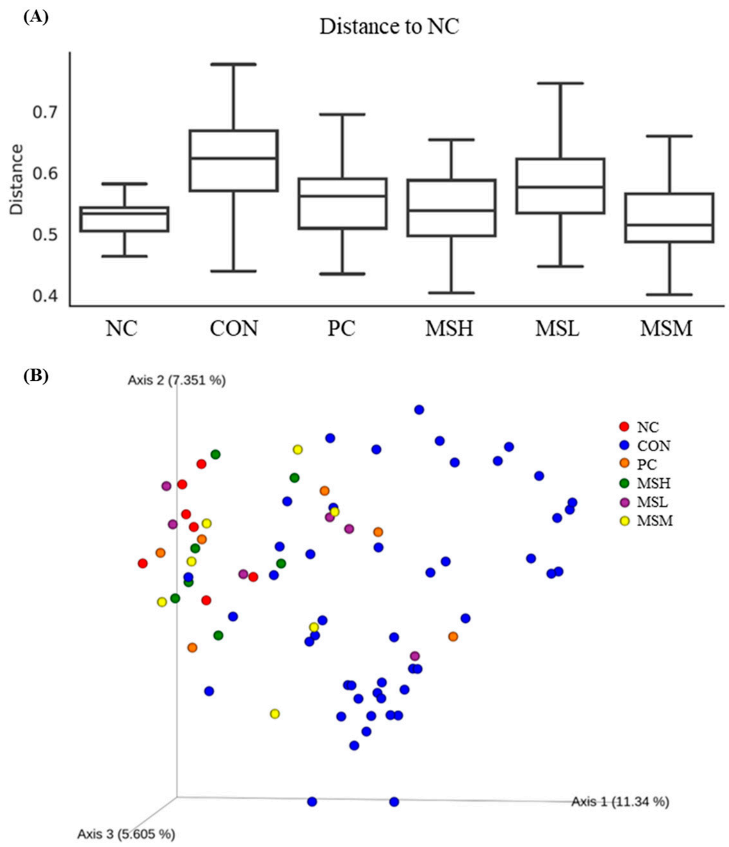


**Supplementary Figure S5.** Plots showing beta diversities across the microbiomes of STZ-induced diabetic mice from different treatment groups. (A) Beta diversity group significance box plot and (B) beta diversity 3D plot.

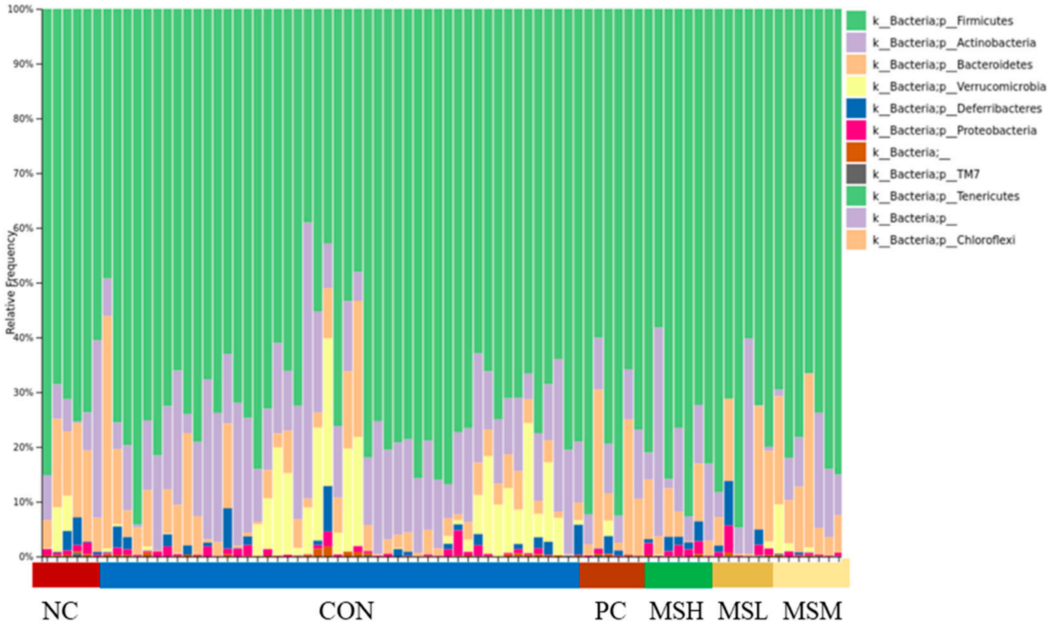


**Supplementary Figure S6.** Bar plots depicting taxonomical annotation (at the phylum level) of the microbiomes of STZ-induced diabetic mice from all six treatment groups.

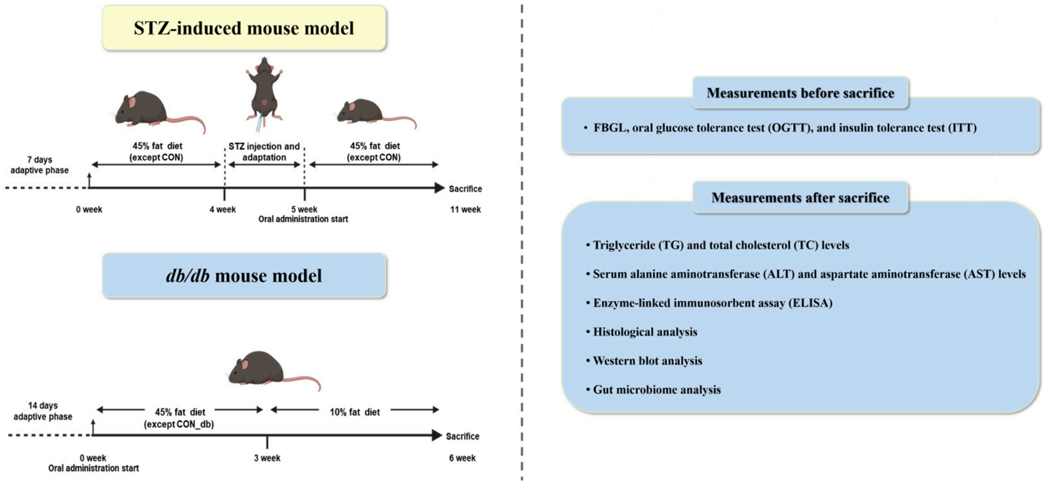


**Supplementary Figure S7.** Experimental design and sampling timeline in this study.
